# Supplementary material for: Mitochondrial Introgression, Color Pattern Variation, and Severe Demographic Bottlenecks in Three Species of Malagasy Poison Frogs, Genus Mantella
Source: Genes (Basel). 2019 Apr 23;10(4):317. doi: 10.3390/genes10040317 (PMC6523892; doi:10.3390/genes10040317)
Supplement: Supplementary file 1 [file genes-10-00317-s001.pdf]

Supplementary Materials

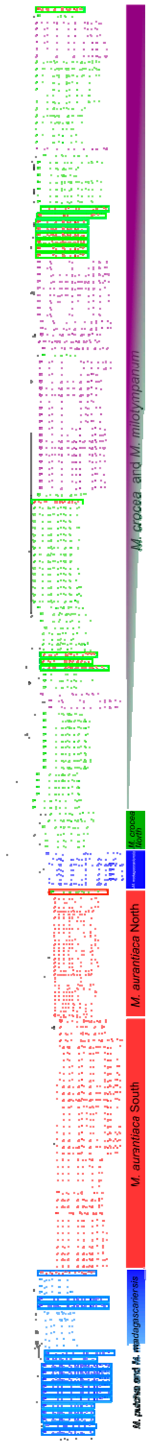

Figure S1. Neighbor-Joining tree reconstructed using the p-distance method and using complete COB dataset, the 17 shorter sequences of *M. aurantiaca* and an homologous sequence of *M. baroni*. Numbers at nodes indicate

bootstrap values. Introgressed samples are marked with a coloured box. Color code as in the haplotype network graphical support.

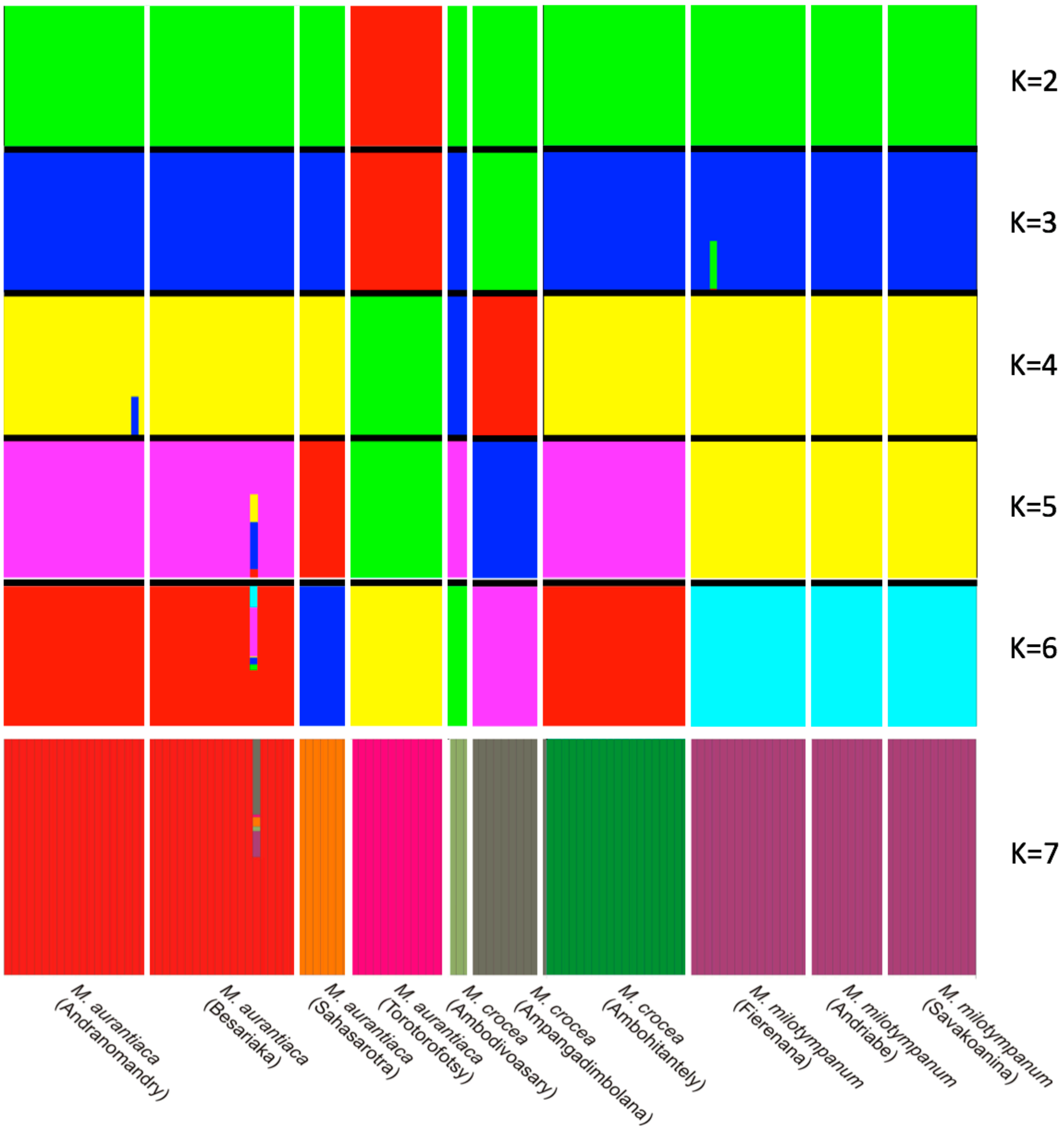

Figure S2. Bayesian analysis of the selected populations of the *Mantella madagascariensis* group used for the analysis of population structure. Barplots of the clustering solution starting from the suboptimal solution for 2 clusters only (K=2) and progressively adding one cluster at the time until the optimal solution of 7 clusters is shown (bottom). Each individual in the analysis is represented by a vertical bar, and groups of individuals of the same sampling site are grouped in boxes with an associated label below and separated by white bars. Admixed individuals appear as those individuals' whose genotype is of more than one colour, with the fraction of each colour indicating the proportion of the genotype assigned to the cluster of that colour. Note that colours assigned too each population are not consistent among the various plots.

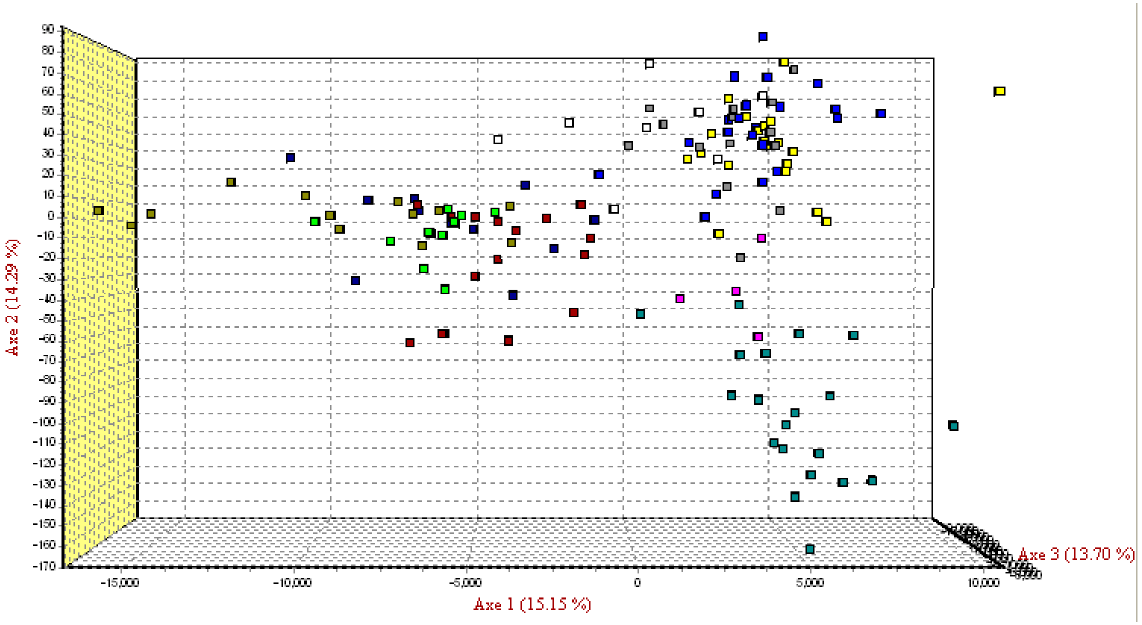

Figure S3. Results of the correspondence factor analysis that shows overlap between localities analysed by a group-based approach.

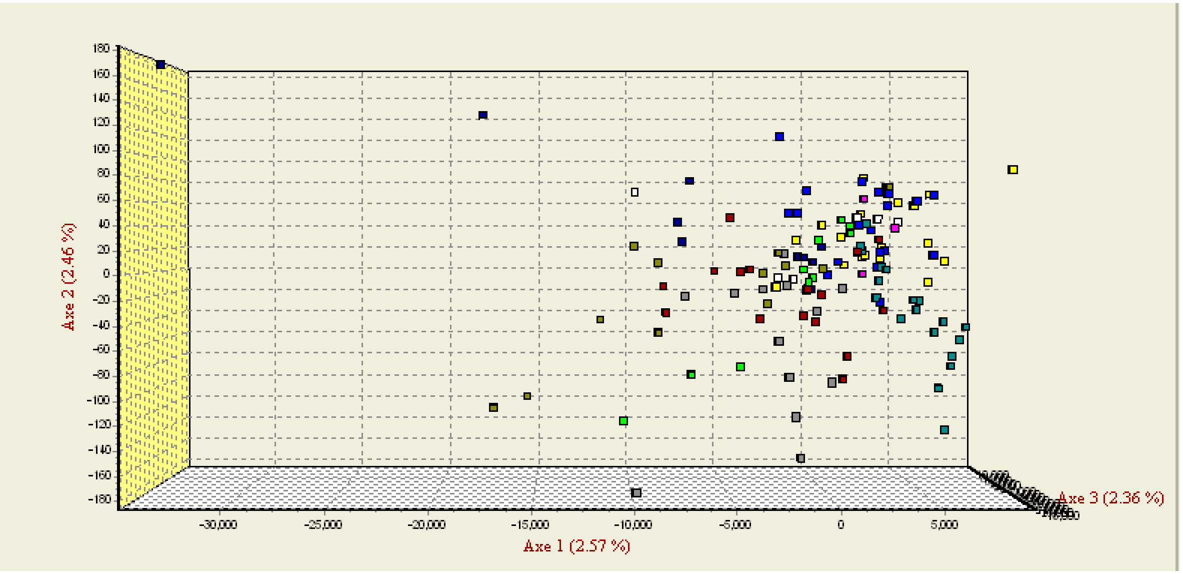

Figure S4. Results of the correspondence factor analysis that shows overlap between the localities analysed by an individual-based approach.

Table S1. Sample ID, field number, taxon, sampling locality and ID, note on the phenotype and respective COB, RAG-1 and RAG-2 haplotypes and the raw scoring for each of the 9 analysed microsatellite. In yellow are highlighted the samples showing sign of introgression. Samples in bold are the one that have been retained for population structure and demographic analyses.

Extraction number	Field number	Species	Locality	Locality code	Phenotype	Cytb	Rag1	Rag2	B2X		A9		G10X		B12X		B7		B8		M14		M10		F12	
AC707	no number	*aurantiaca*	Andranomandry	Amd	Orange translucent	C01	R01,R02	4, 6	161	161	169	197	331	347	334	362	282	378	216	236	144	144			160	184
AC708	no number	*aurantiaca*	Andranomandry	Amd	Orange translucent	C01	R02,R03	4, 7	137	153	173	181	363	371	342	374	282	282	240	240			151	151	160	160
AC709	FG/MV 2002-2055	*aurantiaca*	Andranomandry	Amd	Orange translucent	C02	R01,R02	7, 7							366	390	274	282	216	216	122	122	151	153	148	160
AC710	no number	*aurantiaca*	Andranomandry	Amd	Orange translucent	C01	R04,R05	6, 1	161	161	177	197	379	379	330	338	278	290	224	224	144	144	153	153		
AC711	no number	*aurantiaca*	Andranomandry	Amd	Orange translucent	C01	R01,R06	4, 7	133	153	189	197	363	375	314	346	262	274	240	240	158	158	151	161	164	168
AC712	FG/MV 2002-2054	*aurantiaca*	Andranomandry	Amd	Orange translucent	C01	R02,R07		129	137	165	205	343	351	314	362	290	290	236	240	122	122	153	155	156	160
AC713	FG/MV 2002-2050	*aurantiaca*	Andranomandry	Amd	Orange translucent	C01	R01,R05		133	137	185	189	359	375	338	382	262	274	244	248	112	112	153	155	160	200
AC714	FG/MV 2002-2051	*aurantiaca*	Andranomandry	Amd	Orange translucent	C01	R01,R06		153	173	193	197	359	375	330	354	282	282	216	216	122	122	153	153	160	200
AC715	FG/MV 2002-2052	*aurantiaca*	Andranomandry	Amd	Orange translucent	C01	R08,R05		133	133	173	177	367	367	334	354	250	274	216	248	138	158	151	151	180	196
**AC716**	**FG/MV 2002-2048**	***aurantiaca***	**Andranomandry**	**Amd**	**Orange translucent**	C19	R01,R01		141	161	177	185	371	375	314	326	286	298	200	200	148	148	151	161	156	160
AC717	FG/MV 2002-2049	*aurantiaca*	Andranomandry	Amd	Orange translucent	C01	R05,R03		141	173	193	205	319	351	354	366	278	314	208	208			149	151	152	160
AC718	no number	*aurantiaca*	Andranomandry	Amd	Orange translucent	C01	R05,R05		157	173	177	197	351	375	362	362	282	282	248	248			153	155	160	168
**AC719**	**no number**	***aurantiaca***	**Andranomandry**	**Amd**	**Orange translucent**	C04	R01,R01		133	141	185	189	359	371	342	382	274	282	240	240	112	158			160	180
AC720	FG/MV 2002-2058	*aurantiaca*	Andranomandry	Amd	Orange translucent	C01	R02,R06		141	161	181	205	335	335	342	390	258	278	252	252	148	162	151	151	156	180
AC721	no number	*aurantiaca*	Andranomandry	Amd	Orange translucent	C01	R01,R03		141	161	181	201	339	339	338	362	282	286	240	240	148	148	145	151	152	156
AC722	FG/MV 2002-2059	*aurantiaca*	Andranomandry	Amd	Orange translucent	C01	R01,R02		137	137	189	197	371	371	330	342	274	282	236	236	118	118	149	151	156	168
**AC723**	**FG/MV 2002-2056**	***aurantiaca***	**Andranomandry**	**Amd**	**Orange translucent**	C05	R03,R06		161	173	169	209	335	347	314	358	274	274	208	248	118	122	151	153	180	200
AC724	FG/MV 2002-2053	*aurantiaca*	Andranomandry	Amd	Orange translucent	C01	R08,R03		141	161	185	193	359	371	334	354	262	262	220	220	150	150	151	153	156	196
AC725	no number	*aurantiaca*	Andranomandry	Amd	Orange translucent	C01	R02,R09		137	141	169	177	339	347	378	378	278	282	224	244	122	122	153	161	176	200
AC726	no number	*aurantiaca*	Andranomandry	Amd	Orange translucent	C01	R01,R03		157	173	189	205	363	363	342	362							149	153	160	192
AC727	FG/MV 2002-2057	*aurantiaca*	Andranomandry	Amd	Orange translucent	C01	R04,R03		133	153	193	197	375	379	330	362	282	282	216	248	122	122	151	155	164	168
A4	422	*aurantiaca*	Besariaka	Bes	Orange translucent	C01	R01,R01		133	145	141	225	351	383	354	386	250	290	268	268	130	160	151	151	152	176
A5	414	*aurantiaca*	Besariaka	Bes	Orange translucent	C01	R01,R02		141	149	177	225	371	371	338	358	262	278	220	220	130	138	151	151	164	188
**A6**	**406**	***aurantiaca***	**Besariaka**	**Bes**	**Orange translucent**	C08	R02,R05		133	153	185	205	383	383	346	382	282	298	236	236	136	148	151	151	160	200
**B4**	**421**	***aurantiaca***	**Besariaka**	**Bes**	**Orange translucent**	C04	R01,R10		125	157	193	205	351	367	358	382	262	278					151	153	164	192

B5	413	*aurantiaca*	Besariaka	Bes	Orange translucent	C01	R02,R05		161	169	177	177	311	323	342	394	266	302	268	268	158	158	151	153	164	192
B6	405	*aurantiaca*	Besariaka	Bes	Orange translucent	C01	R01,R11		145	161	177	177	359	363	350	362	262	298	220	220	130	146	151	153	172	176
C4	420	*aurantiaca*	Besariaka	Bes	Orange translucent	C01	R01,R02		137	173	173	173	403	403	346	362	262	266	220	220	138	138	151	153	160	168
C5	412	*aurantiaca*	Besariaka	Bes	Orange translucent	C04	R02,R05		133	133	193	209	371	371	342	342	266	306			106	156	151	151	156	168
C6	404	*aurantiaca*	Besariaka	Bes	Orange translucent	C01	R02,R02		129	141	193	197	355	359	342	398	266	282	216	216	158	158	151	151	188	192
D4	419	*aurantiaca*	Besariaka	Bes	Orange translucent	C04	R05,R12		137	161	177	197	379	383	338	394	258	266	240	240	104	138	153	153	160	200
D5	411	*aurantiaca*	Besariaka	Bes	Orange translucent	C01	R01,R02		133	149	177	217	363	363	330	390	282	302	220	220			151	151	160	200
D6	403	*aurantiaca*	Besariaka	Bes	Orange translucent	C07	R13,R14		137	173	197	205	335	351	342	358	266	266	240	240	144	144	151	151	160	164
E4	418	*aurantiaca*	Besariaka	Bes	Orange translucent	C04	R02,R06		145	145	177	197	363	375	326	366	266	282	236	236	116	136	149	151	180	200
E5	410	*aurantiaca*	Besariaka	Bes	Orange translucent	C01	R08,R06		137	145	193	197	339	359	338	354	258	382	232	240	94	130	151	151	160	188
E6	401	*aurantiaca*	Besariaka	Bes	Orange translucent	C04	R01,R12		125	157	177	193	347	363	354	366	266	282	216	216	120	148	153	159	160	200
F4	417	*aurantiaca*	Besariaka	Bes	Orange translucent	C06	R12,R06		169	169	193	213	375	375	334	342	266	270	256	256	122	136	151	151	160	160
F5	409	*aurantiaca*	Besariaka	Bes	Orange translucent	C04	R04,R02		133	157	193	197	347	371	330	362	246	310	208	208	104	158	159	159	160	160
G4	416	*aurantiaca*	Besariaka	Bes	Orange translucent	C05	R02,R03		129	137	181	209	379	379	350	398	250	286	240	240	122	122	151	159	180	184
G5	408	*aurantiaca*	Besariaka	Bes	Orange translucent	C09	R02,R15		133	173	173	197	363	363	330	378	258	262	228	248	120	158	153	153	192	200
H4	415	*aurantiaca*	Besariaka	Bes	Orange translucent	C01	R02,R16		133	173	193	197	331	347	354	382	270	298			130	160	151	151	160	188
H5	407	*aurantiaca*	Besariaka	Bes	Orange translucent	C07	R02,R17		157	165	181	197	371	383	314	342	266	298	244	244	130	158	151	153	160	192
A3	111	*aurantiaca*	Sahasarotra	Sro	Orange translucent	C01	R18,R18		165	165	189	189	347	367	330	358	274	278	212	224	124	164	151	151	160	176
B3	110	*aurantiaca*	Sahasarotra	Sro	Orange translucent	C01	R02,R19		153	153	201	205	359	359	354	390	290	294	248	248			151	153	160	168
C3	109	*aurantiaca*	Sahasarotra	Sro	Orange translucent	C01	R01,R02		133	133	185	213	347	347	330	358	258	274	212	212	124	124	151	151	160	164
D3	108	*aurantiaca*	Sahasarotra	Sro	Orange translucent	C01	R20,R03		137	161	209	209	363	363	350	362	282	282	216	216	124	144	151	151	164	164
E3	107	*aurantiaca*	Sahasarotra	Sro	Orange translucent	C01	R04,R21		169	173	185	213	363	363	330	366	278	278	264	264	134	134	151	151		
F3	106	*aurantiaca*	Sahasarotra	Sro	Orange translucent	C01	R22,R23		137	165	205	225	359	383	366	370	274	294	216	216	98	138	151	151	160	184
G3	105	*aurantiaca*	Sahasarotra	Sro	Orange translucent	C01	R24,R25		133	177	209	221	351	351	330	354	218	282	216	216	124	124	151	151	160	184
H3	104	*aurantiaca*	Sahasarotra	Sro	Orange translucent	C01	R03,R18		133	157	161	185	363	363	366	374	282	310							160	168
A9	ZCMV1077	*aurantiaca*	Torotorofotsy	Tor	Orange translucent	C10	R02,R02		137	137	157	157	347	347	338	342	262	270	224	224	114	130	151	151	148	156
A10	91_Edouard	*aurantiaca*	Torotorofotsy	Tor	Orange translucent	C10	R26,R03		145	145	189	209	327	327	334	358	302	310			130	156			156	164
A11	24_Edouard	*aurantiaca*	Torotorofotsy	Tor	Orange translucent	C10	R02,R27		177	181	189	201	331	343	358	358	262	262	204	204	102	158			140	160
B9	79_Edouard	*aurantiaca*	Torotorofotsy	Tor	Orange translucent	C10	R02,R28		161	161	165	189	323	343	386	390	250	294			118	150	151	151	188	188
B10	63_Edouard	*aurantiaca*	Torotorofotsy	Tor	Orange translucent	C10	R02,R03		141	185	189	209	327	327	358	358	262	334	220	248	102	102			160	172

B11	16_Edouard	*aurantiaca*	Torotorofotsy	Tor	Orange translucent	C10	R29,R30		177	185	165	201	315	327	342	342	294	294	220	220	130	156	151	151	156	160
C9	22_Edouard	*aurantiaca*	Torotorofotsy	Tor	Orange translucent	C10	R31,R32		137	137	217	221	343	343	338	362	262	274	248	248	126	126			184	188
C10	8_Edouard	*aurantiaca*	Torotorofotsy	Tor	Orange translucent	C10	R02,R03		141	145	185	209	343	343	346	386	262	274	216	232	102	138	151	151	164	164
C11	15_Edouard	*aurantiaca*	Torotorofotsy	Tor	Orange translucent	C10	R02,R03		137	173	161	209	347	347	338	386	274	310	244	244	156	156			156	172
D9	125_Edouard	*aurantiaca*	Torotorofotsy	Tor	Orange translucent	C10	R02,R02		141	185	185	197	363	363	338	358	274	318			118	118	151	151	156	172
D10	27_Edouard	*aurantiaca*	Torotorofotsy	Tor	Orange translucent	C11	R29,R30		141	177	165	213	323	323	390	394	298	310	256	256	108	118				
D11	46_Edouard	*aurantiaca*	Torotorofotsy	Tor	Orange translucent	C10	R03,R03		129	141	189	201			338	362	250	274	220	220	126	126			160	188
E9	3_Edouard	*aurantiaca*	Torotorofotsy	Tor	Orange translucent	C10	R33,R03		121	161	161	213	331	331	342	342	274	286			94	94			156	188
E10	144_Edouard	*aurantiaca*	Torotorofotsy	Tor	Orange translucent	C10	R02,R34		121	161	177	177			342	342	262	262			102	102	151	151	160	180
F9	139_Edouard	*aurantiaca*	Torotorofotsy	Tor	Orange translucent	C10	R02,R16		141	185	165	165	343	347	358	374	262	310	244	244	156	156	151	151	156	160
F10	87_Edouard	*aurantiaca*	Torotorofotsy	Tor	Orange translucent	C10	R03,R16		141	149	201	213	363	363	358	386	262	266			150	158	151	153	160	180
G10	92_Edouard	*aurantiaca*	Torotorofotsy	Tor	Orange translucent	C10	R02,R16		157	161	185	189	311	327	358	386	270	298	248	248	118	118	151	151	164	180
H10	40_Edouard	*aurantiaca*	Torotorofotsy	Tor	Orange translucent	C10	R35,R16		121	169	193	201	339	347	386	390	270	314			156	156	151	151	164	172
B2	64	*crocea*	Ambodivoasary	Adi	green	C12	R04,R30		137	137	197	197	339	339	342	378	294	298	220	240	120	126	149	151	176	180
C2	63	*crocea*	Ambodivoasary	Adi	green	C05	R03,R30		137	141	201	201			346	362	250	286	224	224	120	126	151	153	180	184
D2	62	*crocea*	Ambodivoasary	Adi	green	C12	R01,R03		145	149	177	177			334	342	258	298	244	244			149	151	176	176
E2	61	*crocea*	Ambodivoasary	Adi	green	C12	R03,R03		137	141	193	205	375	375	334	334	258	294	224	224	120	120	149	155	208	208
F2	60	*crocea*	Ambodivoasary	Adi	green	C05	R03,R03		157	165	181	185			334	342	250	270			120	120			160	176
B8	ZCMV1084	*crocea*	Ambohitantely	Ahi	green	C09	R01,R01		145	149	197	197	359	367	374	426	246	282	232	236	140	140	151	153	160	164
B12	no number 2007	*crocea*	Ambohitantely	Ahi	green	C09	R01,R36		141	145	189	193	363	371	346	394	282	286	228	228	106	106	151	153	152	164
C8	ZCMV1083	*crocea*	Ambohitantely	Ahi	green	C09	R37,R37		161	177	185	205	359	367	342	366	270	278	224	224	136	136	149	153	156	156
C12	no number 2007	*crocea*	Ambohitantely	Ahi	green	C17	R01,R28		141	157	201	205	351	355	362	410	282	286	220	232	146	146			128	148
D8	ZCMV1082	*crocea*	Ambohitantely	Ahi	green	C12	R01,R25		137	177	213	213	351	355	350	350	290	330	228	232	146	156	151	153	136	156
D12	no number 2007	*crocea*	Ambohitantely	Ahi	green	C09	R28,R28		157	157	189	205	363	371	334	346	238	238	224	240					140	160
E8	ZCMV1081	*crocea*	Ambohitantely	Ahi	green	C09	R36,R36		149	149	181	181	343	415	334	366	270	282	224	248	138	138			140	156
E12	no number 2007	*crocea*	Ambohitantely	Ahi	green	C09	R28,R28		157	157	197	205	339	379	358	362	282	290	228	248	120	120	151	153	140	168
F8	ZCMV1080	*crocea*	Ambohitantely	Ahi	green	C09	R36,R36		133	133	193	197	331	355	362	370	238	286	208	252	140	140	151	153	164	172
F11	no number 2007	*crocea*	Ambohitantely	Ahi	green	C15	R01,R38		149	149	177	197	343	359	346	346	282	282	220	252	98	98	149	151	140	160
F12	no number 2007	*crocea*	Ambohitantely	Ahi	green	C09	R01,R01		157	161	181	197	355	367	362	362	238	274	228	228	142	142	149	149	140	164
G7	ZCMV1087	*crocea*	Ambohitantely	Ahi	green	C09	R01,R36		153	153	177	205	363	367	362	362	238	238	232	236	120	120	149	149	144	160
G8	ZCMV1079	*crocea*	Ambohitantely	Ahi	green	C09	R01,R28		137	141	189	197	343	403	374	374	282	294	236	244	148	148	149	149	132	160

**G11**	**no number 2007**	***crocea***	**Ambohitantely**	**Ahi**	**green**	C13	R39,R40		**141**	141	**157**	**161**	**367**	**375**	**354**	**362**	**238**	**298**	**208**	**248**	**140**	**140**	**151**	**151**	140	**160**
**G12**	**no number 2007**	***crocea***	**Ambohitantely**	**Ahi**	**green**	C09	R28,R28		**145**	145	**193**	**237**	**359**	**359**	**338**	**358**	**294**	**298**	**220**	**228**	**130**	**140**	**151**	**151**	**156**	**168**
**A8**	**ZCMV1085**	***crocea***	**Ambohitantely**	**Ahi**	**green**	C09	R41,R41		**141**	**141**	**181**	**201**	**359**	**367**	**362**	**374**	**238**	**250**	**204**	**204**			**153**	**153**	**132**	**140**
**A12**	**no number 2007**	***crocea***	**Ambohitantely**	**Ahi**	**green**	C09	R38,R28		**181**	181	**157**	**197**	**347**	**351**	**362**	**370**	**270**	**278**	**232**	**232**			**151**	**151**	**108**	**116**
**H7**	**ZCMV1086**	***crocea***	**Ambohitantely**	**Ahi**	**green**	C13	R01,R36		**153**	157	**165**	**197**	**339**	**355**	**366**	**366**	**238**	**290**	**232**	**240**	**126**	**138**	**149**	**153**	**136**	**140**
**H8**	**ZCMV1078**	***crocea***	**Ambohitantely**	**Ahi**	**green**	C12	R01,R36		**149**	149	**193**	**237**	**367**	**371**	**354**	**358**	**238**	**238**	**208**	**244**	**142**	**142**	**151**	**153**	**156**	**160**
**H11**	**no number 2007**	***crocea***	**Ambohitantely**	**Ahi**	**green**	C09	R01,R28		**145**	145	**181**	**185**	**351**	**407**	**334**	**362**	**218**	**270**	**224**	**256**	**112**	**130**	**149**	**149**		
**H12**	**no number 2007**	***crocea***	**Ambohitantely**	**Ahi**	**green**	C09	R36,R38		**149**	149	**157**	**197**	**355**	**371**	**358**	**402**	**258**	**278**	**228**	**240**					**160**	**160**
**OJ5**	**2007_OJCROC5_07**	***crocea***	**Ambohitantely**	**Ahi**	**green**	C16	R42,R03		149	181	193	225	391	395	338	338	246	262	252	252					164	172
**OJ2**	**2007_OJCROC2_07**	***crocea***	**Ambohitantely**	**Ahi**	**green**	C16	R02,R25		165	177	189	193	359	359	342	342	286	294	244	244					172	180
**OJ1**	**2007_OJCROC1_07**	***crocea***	**Ambohitantely**	**Ahi**	**green**	C16	R02,R03		145	181	193	209	359	367	354	354	290	298	244	248					172	172
**OJ6**	**2007_OJCROC6_07**	***crocea***	**Ambohitantely**	**Ahi**	**green**	C14	R43,R44		173	177	181	185	367	367	302	302			252	260					172	176
**OJ7**	**2007_OJCROC7_07**	***crocea***	**Ambohitantely**	**Ahi**	**green**	C12	R01,R12		161	161	193	213	383	383	370	378	278	278	232	252					172	172
**AC776**	**ZCMV 1028**	***crocea***	**Ampangadimbolana**	**Amp**	**brown**	C20	R45,R03		145	173	153	201	339	363	330	370	270	274	244	244			151	151	184	184
**AC777**	**ZCMV 1029**	***crocea***	**Ampangadimbolana**	**Amp**	**brown**	C12	R04,R04		145	165	197	197	359	407	330	366	286	302					153	153	156	188
**AC778**	**ZCMV 1030**	***crocea***	**Ampangadimbolana**	**Amp**	**brown**	C12	R01,R04		149	149	197	197	359	359	334	350	286	302	236	236			153	153	164	172
**AC779**	**ZCMV 1031**	***crocea***	**Ampangadimbolana**	**Amp**	**brown**	C18	R01,R04		169	169	185	189	375	375	334	362	262	270	248	248	114	114	147	151	184	184
**AC780**	**ZCMV 1032**	***crocea***	**Ampangadimbolana**	**Amp**	**brown**	C12	R46,R47		149	149	189	205	351	351	342	354	262	266			136	136	151	151	180	188
**AC781**	**ZCMV 1033**	***crocea***	**Ampangadimbolana**	**Amp**	**brown**	C12	R48,R49				185	185	363	363	294	354	262	262	296	296	154	154	151	151	172	192
**AC782**	**ZCMV 1034**	***crocea***	**Ampangadimbolana**	**Amp**	**brown**	C20	R48,R50		169	169	197	205	379	379	294	334	262	294	296	296	104	126	151	151	180	184
**AC783**	**ZCMV 1035**	***crocea***	**Ampangadimbolana**	**Amp**	**brown**	C12	R47,R51		145	145	189	205	359	359	342	342			220	220			151	151	184	184
**AC784**	**ZCMV 1036**	***crocea***	**Ampangadimbolana**	**Amp**	**brown**	C12	R04,R50		165	165	177	189	359	375	334	334	270	302	236	256	114	114	147	153	192	192
**AC785**	**ZCMV 1037**	***crocea***	**Ampangadimbolana**	**Amp**	**brown**	C12	R03,R03	11, 11	145	173	197	205	355	363	342	350	262	262	220	220	106	106	149	153	176	188
**AC787**	**ZCMV 1039**	***crocea***	**Ampangadimbolana**	**Amp**	**brown**	C19	R28,R52		149	149	185	185	363	363	334	374	258	294			126	126	147	151	188	188
**AC788**	**ZCMV 1040**	***crocea***	**Ampangadimbolana**	**Amp**	**brown**	C20	R53,R50	12, 13	145	149	189	201	355	355	322	414	258	258	236	236			153	153	180	180
C1	Marovoy gara_94	*crocea*	Ankosy	Ank	brown	C22	R04,R03		141	145	197	205	355	359	334	346	250	306	228	228						
G2	Marovoy gara_93	*crocea*	Ankosy	Ank	brown	C21	R54,R03		133	141	165	185	439	439	346	374	266	290	220	220					184	184
H2	Marovoy gara_92	*crocea*	Ankosy	Ank	brown	C05	R55,R03		141	145	205	209			338	346	250	290	224	224					172	188
AC1068	ZCMV11245	*crocea*	Hevirina-Makira	Hev	green	C23	NA		121	137	173	181	371	435	350	354	266	278	232	240					156	164
AC1069	ZCMV11246	*crocea*	Hevirina-Makira	Hev	green	C26	R56,R56		121	125	185	189	367	383	374	374	278	298	224	240					156	156
AC1070	DRV05866	*crocea*	Hevirina-Makira	Hev	green	C26	NA		125	125	181	237	347	371	334	362	266	270	228	228					156	156
AC1071	DRV05867	*crocea*	Hevirina-Makira	Hev	green	C24	R57,R57		125	133	173	213	367	383	326	398	254	270	232	240					136	140

AC1072	DRV05868	*crocea*	Hevirina-Makira	Hev	green	C26	R57,R57		133	141	217	229	379	415	366	370			236	236		164	172
AC1073	DRV05869	*crocea*	Hevirina-Makira	Hev	green	C25	NA		133	133	161	161	371	435	350	350	266	278	224	236		164	164
OJ10	2008_OJ10_08	*crocea*	Prolemur site, Andasibe region	Asi	brown	C18	R03,R03		137	173	161	201	343	343	342	362	278	298	212	228		164	188
OJ3	2008_OJ3_08	*crocea*	Prolemur site, Andasibe region	Asi	brown	C27	R03,R03						383	383								156	164
OJ2	2008_OJ2_08	*crocea*	Prolemur site, Andasibe region	Asi	brown	C18	R05,R03		129	141	165	181	343	367	338	338	246	278	224	240		164	164
OJ1	2008_OJ1_08	*crocea*	Prolemur site, Andasibe region	Asi	brown	C28	R04,R04		141	153	181	209	343	379	338	338	262	278	228	228		168	192
KCW12	2008_KCW12_08	*crocea*	Prolemur site, Andasibe region	Asi	brown	C27	R01,R04		137	153	181	197	379	383	322	374	234	278	224	256		168	176
KCW11	2008_KCW11_08	*crocea*	Prolemur site, Andasibe region	Asi	brown	C28	R03,R03		129	141	193	209	339	367	330	338	188	188	164	172		176	180
KCW10	2008_KCW10_08	*crocea*	Prolemur site, Andasibe region	Asi	brown	C28	R03,R03		153	153	189	225	383	383	338	370	258	278	228	228		172	172
KCW9	2008_KCW9_08	*crocea*	Prolemur site, Andasibe region	Asi	brown	C27	R04,R03		157	157	193	209	347	383	338	366	262	278	252	252		160	164
KCW8	2008_KCW8_08	*crocea*	Prolemur site, Andasibe region	Asi	brown	C27	R03,R03		145	153	181	217	339	351	322	338	258	270	256	260		164	164
KCW7	2008_KCW7_08	*crocea*	Prolemur site, Andasibe region	Asi	brown	C27	R01,R04		129	161	181	193	379	379	322	338	238	290	224	224		164	164
KCW67	2008_KCW6_7_08	*crocea*	Prolemur site, Andasibe region	Asi	brown	C05	R03,R03		141	153	161	169	343	367	338	338			220	256		188	188
KCW66	2008_KCW6_6_08	*crocea*	Prolemur site, Andasibe region	Asi	brown	C27	R04,R03		137	137	177	193	379	391	338	338	278	278	228	228		164	164
KCW65	2008_KCW6_5_08	*crocea*	Prolemur site, Andasibe region	Asi	brown	C27	R04,R30		157	165	181	185	343	371	322	406	234	262	220	220		172	188
KCW64	2008_KCW6_4_08	*crocea*	Prolemur site, Andasibe region	Asi	brown	C27	R03,R03		141	141	185	197	411	411	322	370	278	278	224	256		156	176
KCW61	2008_KCW6_1_08	*crocea*	Prolemur site, Andasibe region	Asi	brown	C27	R04,R04						339	351	350	350	258	278	248	248		184	184
KCW4	2008_KCW4_08	*crocea*	Prolemur site, Andasibe region	Asi	brown	C28	R04,R03		141	141	177	197	343	351	330	338	286	286	236	252		180	180
AC1211	no number	*crocea*	Zahamena	Zah	green	C29	R58,R59		133	133	189	189	363	367	330	350	266	266	220	248		132	180
AC1212	no number	*crocea*	Zahamena	Zah	green	C30	R48,R59		153	153	161	161	363	363	342	346	250	266	220	220		148	180
AC1213	no number	*crocea*	Zahamena	Zah	green	C30	R60,R61		157	157	185	185	363	363	342	346	338	358	220	220		160	188
AC690	GA 2003/206-207_Nr.2	*crocea*	Andaingo (former North of Fierenana)	And	green	C36	R01,R63		137	169	185	193	359	359	334	358	246	378	248	248			
AC790	ZCMV 1056	*madagascariensis*	Besariaka	Bes	_	C42	R19,R21	4, 14	149	153	197	197			354	354	254	270	216	216		164	164
AC791	ZCMV 1057	*madagascariensis*	Besariaka	Bes	_	C43	R19,R19	15, 16	141	153	173	189			338	338	254	274	204	216		144	160
AC792	ZCMV 1058	*madagascariensis*	Besariaka	Bes	_	C42	R19,R18	17, 16	153	153	169	189			334	334	286	374	216	216		148	168
AC795	ZCMV 1061	*madagascariensis*	Besariaka	Bes	_	C42	R18,R18	17, 17	153	153	169	189			314	362	302	302	216	216		148	152
AC796	ZCMV 0915	*madagascariensis*	Besariaka	Bes	_	C44	R21,R18		149	149	173	185			338	342	266	334	204	204		152	164
AC797	ZCMV 0916	*madagascariensis*	Besariaka	Bes	_	C42	R67,R18		141	141	173	193			354	370	254	306	216	220		156	160
AC798	ZCMV 0917	*madagascariensis*	Besariaka	Bes	_	C42	R19,R19		145	153	169	169			338	362	266	270	244	244		152	164
AC799	ZCMV 0918	*madagascariensis*	Besariaka	Bes	_	C42	R21,R18		145	153	185	193			342	366	250	258	216	216		144	152
AC800	ZCMV 0919	*madagascariensis*	Besariaka	Bes	_	C44	R67,R68		153	157	189	189			350	350	250	258	216	216		156	164

G6	FG/MV 2002-0261	*madagascariensis*	Ranomafana	Ran	_	C41	R69,R69		129	157	181	189			358	358	258	266	216	216					100	100
ZMA20329	ZMA20329	*madagascariensis*	Ranomafana	Ran	_	C41	R69,R69		173	189	165	173			354	370	258	258	192	200						
ZMA20328	ZMA20328	*madagascariensis*	Ranomafana	Ran	_	C41	R04,R05										206	258	208	208					168	184
ZMA20327	ZMA20327	*madagascariensis*	Ranomafana	Ran	_	C41	R01,R12		133	133	161	177					258	270	208	208						
ZMA20289	ZMA20289	*madagascariensis*	Ranomafana	Ran	_	C41	R70,R71								350	350			204	204						
OJRF15	2007_OJRF15	*madagascariensis*	Ranomafana	Ran	_	C41	R72,R69		141	145	177	185			350	350	258	270	208	208						
OJRF13	2007_OJRF13	*madagascariensis*	Ranomafana	Ran	_	C41	R69,R69		133	141	169	181			342	354	258	270	208	208						
AC741	2002 F_11_1084	*madagascariensis*	Unknown	Unk	_	C45	R67,R19		137	141	177	185	343	351	346	354	266	274	220	220						
AC742	2002 F_12_1085	*madagascariensis*	Unknown	Unk	_	C43	R73,R02		141	161	173	197	347	423	346	362	258	334	224	224					144	168
AC743	2002 F_13_1086	*madagascariensis*	Unknown	Unk	_	C42	R02,R19		129	153	161	189	355	355	330	330	254	266	216	216					140	156
AC744	2002 F_14_1087	*madagascariensis*	Unknown	Unk	_	C42	R21,R21		133	133	181	181	343	351	338	350	274	334	200	244					148	160
AC745	2002 F_15_1088	*madagascariensis*	Unknown	Unk	_	C42	R65,R19		157	165	189	213	363	387	338	342	270	306	220	220					148	160
AC746	2002 F_16_1089	*madagascariensis*	Unknown	Unk	_	C42	R01,R03		137	141	177	197	327	343	334	346	254	314	220	220					144	160
**AC729**	**2002 A_32**	***milotympanum***	**Fierenana-Sahamarolambo**	**Sah**	**red**	C31	R02,R03	4, 8	173	173	181	189	359	367	350	354	266	266	248	248			145	153	148	180
**AC730**	**2002 A_33**	***milotympanum***	**Fierenana-Sahamarolambo**	**Sah**	**red**	C31	R04,R62	4, 4	181	193	173	173	359	359	354	362	246	290	228	228			153	153	176	180
**AC731**	**2002 A_34**	***milotympanum***	**Fierenana-Sahamarolambo**	**Sah**	**red**	C40	R25,R03	4, 4	141	177	189	193	331	331	354	354	286	290	236	256	128	128	153	155	168	172
**AC732**	**2002 A_35**	***milotympanum***	**Fierenana-Sahamarolambo**	**Sah**	**red**	C12	R02,R03	4, 9	141	145	185	189	351	351	302	382	246	262	216	220	114	114	151	151	148	180
**AC733**	**2002 A_36**	***milotympanum***	**Fierenana-Sahamarolambo**	**Sah**	**red**	C12	R03,R03	3, 10	157	173	169	193	351	371	370	386	282	290	244	244	128	128	151	151	172	180
**AC734**	**2002 A_37**	***milotympanum***	**Fierenana-Sahamarolambo**	**Sah**	**red**	C34	R63,R03		169	185	185	189	359	359	338	358	254	262	248	248	128	128	151	151	152	164
**AC735**	**2002 A_39**	***milotympanum***	**Fierenana-Sahamarolambo**	**Sah**	**red**	C31	R01,R63		137	157	193	197	343	375	342	362	266	266	228	228	124	124	147	153	176	180
**AC736**	**2002 A_40**	***milotympanum***	**Fierenana-Sahamarolambo**	**Sah**	**red**	C31	R03,R03		193	205	189	193	351	411	354	374	250	282	232	232	114	126	147	155	164	164
**AC737**	**2002 A_41**	***milotympanum***	**Fierenana-Sahamarolambo**	**Sah**	**red**	C31	R03,R12		169	169	181	193	359	363	366	366	246	278	252	252	128	134	147	155	140	144
**AC738**	**2002 A_42**	***milotympanum***	**Fierenana-Sahamarolambo**	**Sah**	**red**	C31	R01,R12		165	181	181	193	355	367	338	370	250	262	236	236	102	102	149	149	168	180
**AC747**	**2461**	***milotympanum***	**Fierenana-Sahamarolambo**	**Sah**	**red**	C12	R21,R18		153	169	181	197	355	367	370	382	262	286	252	252	128	128	149	151	148	168
**AC748**	**2462**	***milotympanum***	**Fierenana-Sahamarolambo**	**Sah**	**red**	C31	R01,R03		141	141	173	229	367	371	374	374	250	262	212	248	102	102	153	153	180	180
**AC749**	**2463**	***milotympanum***	**Fierenana-Sahamarolambo**	**Sah**	**red**	C31	R03,R03		137	173	189	213	339	347	350	362	266	290	240	240	94	106	151	153	180	184
**AC750**	**2464**	***milotympanum***	**Fierenana-Sahamarolambo**	**Sah**	**red**	C31	R03,R03		165	169	189	209	343	355	350	370	246	266	220	240	134	134	151	153	168	180
**AC752**	**2466**	***milotympanum***	**Fierenana-Sahamarolambo**	**Sah**	**red**	C33	R02,R02		169	173	189	189	359	363	302	358	246	262	248	248	128	134	151	151	164	180
**AC753**	**2467**	***milotympanum***	**Fierenana-Sahamarolambo**	**Sah**	**red**	C34	R03,R03		173	173	185	189			302	382	246	266			128	128	149	151	176	180
**AC754**	**2481**	***milotympanum***	**Fierenana-Sahamarolambo**	**Sah**	**red**	C12	R01,R03		157	165	185	197	363	379	346	350	270	278	240	264			147	155	156	164
OJBlue	2006_OJBlue	*milotympanum*	Ambatombolana	Abo	unknown	C35	NA		145	153	193	209	335	367	342	358	246	282	256	256					164	180
OJBlue2	2007_OJBlue2	*milotympanum*	Ambatombolana	Abo	unknown	C35	R02,R03		141	149	193	209	343	375	322	338	246	282	256	256					164	180

AC683	2003_Nr.2	*milotympanum*	Andriambe	Abe	red	C35	R01,R64	2, 3	137	145	201	213	359	375	322	370	266	290	252	256	122	144	153	153	176	184
AC684	2003_Nr.1	*milotympanum*	Andriambe	Abe	red	C35	R01,R64	2, 3	149	149	201	213	359	367	322	370	266	290	252	252	122	144	151	151	164	172
AC686	2003_Nr.4	*milotympanum*	Andriambe	Abe	red	C37	R01,R64		185	185	181	201	347	347	346	362	262	294	260	260	108	108	151	153	168	172
AC692	2003_Nr. B13	*milotympanum*	Andriambe	Abe	red	C35	R03,R03		149	169	185	209	383	383	342	346	274	290	220	244	122	140	155	155	180	180
AC693	2003_Nr. B14	*milotympanum*	Andriambe	Abe	red	C35	R01,R01		133	145	225	225	351	383	346	378	254	282	220	220					164	164
AC696	2003_Nr. B11	*milotympanum*	Andriambe	Abe	red	C35	R01,R03		153	161	181	229	359	375	342	354	282	282	256	256	144	144	155	155	164	184
AC699	2003_Nr. B12	*milotympanum*	Andriambe	Abe	red	C35	R01,R04		157	157	189	209	339	347	302	346	278	282	220	220	144	144	147	153	168	180
AC701	2003_Nr. B10	*milotympanum*	Andriambe	Abe	red	C35	R01,R01		145	149	205	213	371	371	322	346	266	294	256	256	122	144	153	155		
AC703	2003_Nr. B15	*milotympanum*	Andriambe	Abe	red	C39	R01,R65		177	185	173	189	347	411	346	362	286	286	220	260	150	150	151	155	172	184
AC706	2003_Nr. B9	*milotympanum*	Andriambe	Abe	red	C35	R64,R03		141	153	189	205	355	367	346	350	274	294	256	256			153	155	164	164
AC728	2003_Nr.3	*milotympanum*	Andriambe	Abe	red	C38	R01,R64		153	185	181	201	347	347	350	366	262	294	260	260	108	120	151	155	168	168
AC681	2003_Nr.3	*milotympanum*	Savakoanina	Sav	green	C12	R02,R12	1, 2	137	149	169	189	375	391	330	378	274	278	248	248			149	149		
AC682	2003_Nr.1	*milotympanum*	Savakoanina	Sav	green	C35	R01,R02		161	161	185	193	355	387	302	358	270	282	248	252					152	168
AC685	2003_Nr.6	*milotympanum*	Savakoanina	Sav	yellow	C35	R01,R01	4, 5			185	189	391	391	358	358	258	262	244	252	108	108	147	153	148	172
AC687	2003_Nr.5	*milotympanum*	Savakoanina	Sav	yellow	C35	R01,R01		149	153	185	189	391	391	358	358	258	262	244	252	108	108	147	153	148	172
AC688	2003_Nr.2	*milotympanum*	Savakoanina	Sav	green	C35	R01,R02		161	161	185	193	355	387	302	358	270	282	252	256	122	122	149	149	152	172
AC689	2003_Nr.4	*milotympanum*	Savakoanina	Sav	green	C12	R02,R12		133	145	165	189	379	391	354	354	266	278	240	244	108	108	149	157	172	176
AC694	2003_Nr. B2	*milotympanum*	Savakoanina	Sav	green	C35	R01,R04		153	153	185	189	391	391	302	302	278	302	240	260			153	153	164	164
AC695	2003_Nr. B3	*milotympanum*	Savakoanina	Sav	green	C35	R01,R02		165	185	185	189	363	375	302	362	278	302	228	248	104	104	149	153	172	172
AC697	2003_Nr. B4	*milotympanum*	Savakoanina	Sav	green	C35	R01,R02		157	157	185	193	383	383	338	378	274	278	224	228	96	96	153	155	152	176
AC698	2003_Nr. B1	*milotympanum*	Savakoanina	Sav	orange	C40	R01,R03		157	185	189	201	363	363	334	358	258	282	232	256	94	116	151	153	176	180
AC700	2003_Nr. B7	*milotympanum*	Savakoanina	Sav	green	C35	R01,R03		133	149	169	201	359	371	354	366	262	262	252	268	106	114	151	153	164	168
AC702	2003_Nr. B6	*milotympanum*	Savakoanina	Sav	green	C35	R01,R03		165	169	201	217	363	387	302	342	274	318	236	256	126	134	155	155	148	172
AC704	2003_Nr. B5	*milotympanum*	Savakoanina	Sav	green	C12	R03,R66		153	153	173	205	367	391	330	342	266	274	244	252	100	158	149	153	152	172
AC705	2003_Nr. B8	*milotympanum*	Savakoanina	Sav	green	C35	R04,R12		157	165	177	201	355	363	362	366	270	306	220	220			153	153	164	180
A2	Andohan 'i Sity_117	*pulchra*	Anala	Ana	_	C47	R65,R74		141	141					362	362	238	326	204	204					140	144
A7	ZCMV2509	*pulchra*	AnAla	Ana	_	C44	R75,R30		161	169	177	193			346	350	238	262	248	256					144	156
C7	ZCMV2490	*pulchra*	AnAla	Ana	_	C44	R74,R74		133	133	153	181			334	346	258	266	220	232					152	156
D1	Andohan 'i Sity_120	*pulchra*	Anala	Ana	_	C46	R74,R74		161	173	153	153			358	374	258	258	204	224					152	164
D7	ZCMV2489	*pulchra*	AnAla	Ana	_	C47	R30,R76		173	181	189	189			354	362	258	274	224	224					160	168
E7	ZCMV2488	*pulchra*	AnAla	Ana	_	C44	R74,R30		169	177	177	177	351	383	366	374	266	278	220	220					152	160
F7	ZCMV2487	*pulchra*	AnAla	Ana	_	C49	R77,R76		145	181	177	185			334	342	266	266	192	204					160	168

H1	Andohan 'i Sity_119	*pulchra*	Anala	Ana	_	C46	R16,R16		161	173	153	193			358	374	258	258	204	204					152	168
H6	ZCMV 2513	*pulchra*	AnAla	Ana	_	C48	R78,R75		141	181	161	177	343	347			238	258	224	224					152	164

Table S2. Taxon, locality names and ID, geographical location and number of individuals sequenced for the COB, RAG-1 and RAG-2 fragments per population. (N), number of samples; (S), number of polymorphic sites (COB length= 552 nucleotides; RAG-1 length= 732 nucleotides; RAG-2 length= 531 nucleotides); (Hd), haplotype diversity; (π), Nei's (1987) nucleotide diversity; (D), Tajima's D. Statistics computed separately for the COB, RAG-1 and RAG-2 gene fragments. 1) *M. aurantiaca*, 2) *M. crocea*, 3) *M. milotympanum*, 4) *M. madagascariensis*, 5) *M. pulchra*.

Taxon	Locality	Code	Lat	Long	COB					RAG-1					RAG-2				
					N	S	Hd	π	D	N	S	Hd	π	D	N	S	Hd	π	D
1	Andranomandry	AMD	-19.03944	48.17611	21	32	0.352	0.013	-0.84	42	14	0.848	0.05	0.15	10	4	0.78	0.004	1.53
	Besariaka forest	BES	-19.03500	48.15250	21	34	0.752	0.027	1.99	42	24	0.848	0.006	-0.68					
	Sahasarotra forest	SRO	-19.17610	48.44030	8	0	0.000	0	nc	16	15	0.958	0.007	0.65					
	Torotorofotsy	TOR	-18.87472	48.37250	18	31	0.111	0.006	-2.49	36	14	0.838	0.006	0.85					
2	Ambodivoasary	ADI	-18.79310	48.29153	5	5	0.60	0.005		10	9	0.644	0.004						
	Ambohitantely	AHI	-18.17825	47.29043	26	35	0.65	0.006	-2.29	52	13	0.864	0.005	0.52					
	mpangadimbolana	AMP	-18.97375	48.08063	12	3	0.64	0.001	-0.83	24	24	0.931	0.009	0.34	4	9	0.83	0.009	-0.154
	Ankosy	ANK	-18.64265	48.28095	3	7	1.0	0.009		6	8	0.800	0.004						
	Hevirina-Makira	HEV	-15.4490	49.1119	6	6	0.8	0.006		6	1	0.533	0.001						
	Andasibe	ASI	-18.76889	48.42521	16	11	0.64	0.007		32	10	0.599	0.003						
	Zahamena	ZAH	-17.6370	48.7680	3	1	0.667	0.001		6	13	0.933	0.009						
	Andaingo	AND	-18.26944	48.48417	1	NC	NC	NC		2	3	1.000	0.004						
3	Fierenana-Sahamarolambo	SAH	-18.53963	48.44547	17	10	0.684	0.004	-0.86	34	16	0.733	0.004	-1.1	10	4	0.667	0.002	-0.339
	Ambatombolana	ABO	-18.60703	48.37305	2	0	0.000	0		2	3	1.000	0.004						
	Andriambe	ABE	-18.61280	48.32611	11	7	0.491	0.003	-1.46	22	4	0.693	0.001	-0.39	4	3	0.667	0.004	2.01
	Savakoanina	SAV	-18.61222	48.40833	14	3	0.473	0.002	-0.24	28	10	0.759	0.003	-0.82	4	3	1.000	0.003	-0.75
4	Besariaka forest	BES	-19.03500	48.15250	9	4	0.556	0.002		18	11	0.778	0.005		8	10	0.857	0.008	
	Ranomafana	RAN	-21.24868	47.37178	7	0	0.000	0		14	13	0.769	0.006						
	Unknown	UNK	NA	NA	6	4	0.600	0.002		12	12	0.924	0.007						
5	An'Ala	ANA	-18.91926	48.48796	9	4	0.861	0.002		18	11	0.863	0.004						

Table S3. List of frequencies of different haplotypes (numbered as in the haplotype networks) per population. Note that for nuclear genes (RAG-1, RAG-2), numbers refer to phased sequences, i.e., two haplotypes were counted per sequenced individual.

	**COB**	**RAG-1**	**RAG-2**
M. aurantiaca			
Andranomandry	C01(17), C02(1), C04(1), C05(1), C19(1)	R01(12), R02(7), R03(7), R04(2), R05(6), R06(4), R07(1), R08(2), R09(1)	1(1), 4(3), 6(2), 7(4)
Besariaka forest	C01(9), C04(6), C05(1), C06(1), C07(2), C08(1), C09(1)	R01(8), R02(14), R03(1), R04(1), R05(4), R06(3), R08(1), R10(1), R11(1), R12(3), R13(1), R14(1), R15(1), R16(1), R17(1)	
Sahasarotra forest	C01(8)	R01(1), R02(2), R03(2), R04(1), R18(3), R19(1), R20(1), R21(1), R22(1), R23(1), R24(1), R25(1)	
Torotorofotsy	C10(17), C11(1)	R02(12), R03(8), R16(4), R26(1), R27(1), R28(1), R29(2), R30(2), R31(1), R32(1), R33(1), R34(1), R35(1)	
M. crocea			
Ambodivoasary	C05(2),C12(3)	R01(1), R03(6), R04(1), R30(2)	
Ambohitantely	C09(15), C12(3),C13(2), C14(1), C15(1), C16(3), C17(1)	R01(14), R02(2), R03(2), R12(1), R25(2), R28(10), R36(9), R37(2), R38(3), R39(1), R40(1), R41(2), R42(1), R43(1), R44(1)	
Ampangadimbolana	C12(7), C18(1), C19(1), C20(3)	R01(2), R03(3), R04(5), R28(1), R45(1), R46(1), R47(2), R48(2), R49(1), R50(3), R51(1), R52(1), R53(1)	11(2), 12(1), 13(1)
Ankosy	C05(1), C21(1), C22(1)	R03(3), R04(1), R54(1), R55(1)	
Hevirina-Makira	C23(1), C24(1), C25(1), C26(3)	R56(2), R57(4)	
Andasibe	C05(1), C18(2), C27(9), C28(4)	R01(2), R03(18), R04(10), R05(1), R30(1)	
Zahamena	C29(1), C30(2)	R48(1), R58(1), R59(2), R60(1), R61(1)	
Andaingo	C36(1)	R01(1), R65(1)	
M. milotympanum			
Fierenana-Sahamarolambo	C12(4), C31(9), C33(1), C34(2), C40(1)	R01(4), R02(4), R03(17), R04(1), R12(2), R18(1), R21(1), R25(1), R62(1), R63(2)	3(1), 4(6), 8(1), 9(1), 10(1)
Ambatombolana	C35(2)	R01(1), R03(1)	
Andriambe	C35(8), C37(1), C38(1), C39(1)	R01(11), R03(4), R04(1), R64(5), R65(1)	2(2), 3(2)
Savakoanina	C12(3), C35(10), C40(1)	R01(12), R02(6), R03(4), R04(2), R12(3), R66(1)	1(1), 2(1), 4(1), 5(1)
M. madagascariensis			
Besariaka forest	C42(6), C43(1), C44(2)	R18(6), R19(6), R21(3), R67(2), R68(1)	4(1), 14(1), 15(1), 16(2), 17(3)
Ranomafana	C41(7)	R01(1), R04(1), R05(1), R12(1), R69(7), R70(1), R71(1), R72(1)	
Unknown	C42(4), C43(1), C45(1)	R01(1), R02(2), R03(1), R19(3), R21(2), R65(1), R67(1), R73(1)	
M. pulchra			
An'Ala	C44(3), C46(2), C47(2), C48(1), C49(1)	R16(2), R30(3), R65(1), R74(6), R75(2), R76(2), R77(1), R78(1)	

Table S4. Primer name and sequences and PCR conditions used in the present study. PCR conditions start with temperature (in °C) of each step followed by the time in seconds.

Gene	Primer name	Sequence (5'-3')	PCR conditions
**Cytb**	Cytb-c	CTACTGGTTGTCCTCCGATTCATGT	94(90s) [94(30), 51(45), 72(90)x35] 72(600)
**Cytb**	CBJ 10933	TATGTTCTACCATGAGGACAAATATC	
**RAG-1**	RAG1-FI	CAGTGACCTSATGCTGGAGA	94(120s) [94(20), 60(50), 72(180)x39] 72(600)
**RAG-1**	RAG1-RIII	TCAATGATCTCTGGGACGTG	
**RAG-2**	RAG2-FII	GBGAGGTCAGYCAGTATCTC	94(120s) [94(20), 60(50), 72(180)x39] 72(600)
**RAG-2**	RAG2-RI	AGCGCTYGAGACAGAGAGGT	
**BDNF**	BDNF-F1	ACCATCCTTTTCCTKACTATGG	95(120s) [94(35), 53(45), 72(120)x39] 72(600)
**BDNF**	BDNF-R1	CTATCTTCCCCTTTTAATGGTC	
**12S rRNA**	12Sa-L	AAACTGGGATTAGATACCCCACTAT	94(90s) [94(45), 55(45), 72(90)x32] 72(300)
**12S rRNA**	12Sb-H	GAGGGTGACGGGCGGTGTGT	
**16S rRNA**	16Sa-L	AAACTGGGATTAGATACCCCACTAT	94(90s) [94(45), 55(45), 72(90)x32] 72(300)
**16S rRNA**	16Sb-H	CCGGTCTGAACTCAGATCACGT	
**16S rRNA**	16SL3	AGCAAAGAHYWWACCTCGTACCTTTTGCAT	94(90s) [94(45), 55(45), 72(90)x32] 72(300)
**16S rRNA**	16SR3	AGCAAAGAHYWWACCTCGTACCTTTTGCAT	
**Cox1**	CO1-Chmf4	TYTCWACWAAYCAYAAAGAYATCGG	94(90s) [94(30), 49(45), 72(90)x35] 72(600)
**Cox1**	CO1-Chmr4	ACYTCRGGRTGRCCRAARAATCA	

Table S5. Prior distributions of the parameters estimated in Msvar. The parameters shown in the table are the current effective population size (N0), the ancestral effective population size (Nt), the mutation rate (mu) and the time of the change in effective population size (t). For each parameter the mean of the distribution (e.g. N0) and its associated variance (e.g. V(N0)) are shown. Numbers in the table are log values as used by Msvar, e.g. N0 = 3 corresponds to 103. The priors for the bottleneck scenarios are shown as Bot 1-3, the priors of the expansion scenarios as Exp 1-3, and the priors of the sable demography as Sta 1-3. The hyperprior distribution for each parameter had a mean of means across loci equal to the prior mean, and a variance across means equal to the variance of the prior (e.g. for Bot 1 the hyperpriors mean of means of N0 was 3, and variance of means was 1). The variance of means was set to 0 and the variance of variances to 0.5 for all parameters.

**Model**	**N0**	**V(N0)**	**Nt**	**V(Nt)**	**mu**	**V(mu)**	**t**	**V(t)**
Bot 1	3	1	4	1	-3,5	1	4	1
Bot 2	3	1	5	1	-3	1	4	1
Bot 3	3	1	5	1	-3	1	5	1
Exp 1	5	1	3	1	-3	1	4	1
Exp 2	5	1	3	1	-3	1	5	1
Exp 3	5	1	4	1	-3.5	1	4	1
Sta 1	3	1	3	1	-3.5	1	4	1
Sta 2	4	1	4	1	-3.5	1	5	1
Sta 3	5	1	5	1	-3	1	4	1

Table S6. Highest Posterior Density intervals and mode of posterior distributions of demographic parameters in Msvar. S: Scenario; B: Bottleneck; E: Expansion; S: Stable demography; LB and UP are the lower and upper bound intervals of the highest density posterior distribution; N0: current effective population size; Nt: ancestral effective population size; t: time of the bottleneck. Values in the table are log values, e.g. 2 corresponds to 100. The average values fore the mode are highlighted in black.

Taxon	Locality	S	N0			Nt			t		
			95% LB	95% UB	Mode	95% LB	95% UB	Mode	95% LB	95% UB	MODE
M. aurantiaca	Andranomandry	B	1.26	2.41	1.81	4.52	5.59	5.04	1.44	2.57	2.04
		E	1.12	2.56	1.62	4.49	5.57	5.01	1.29	2.64	1.83
		S	1.28	2.46	1.72	4.58	5.63	5.10	1.45	2.58	1.95
					**1.72**			**5.05**			**1.94**
	Besariaka	B	1.54	2.83	2.27	4.59	5.61	5.19	1.47	2.77	2.13
		E	1.54	2.90	2.23	4.60	5.64	5.18	1.48	2.89	2.29
		S	1.74	2.87	2.27	4.61	5.71	5.25	1.72	2.90	2.32
					**2.26**			**5.21**			**2.25**
	Sahasarotra	B	1.70	3.19	2.49	4.74	5.85	5.29	2.00	3.44	2.73
		E	1.90	3.53	2.67	4.81	5.93	5.35	2.22	3.63	2.82
		S	1.73	3.44	2.72	4.80	5.95	5.41	2.09	3.67	2.97
					**2.63**			**5.35**			**2.84**
	Torotorofotsy	B	1.48	2.71	2.15	4.54	5.56	5.11	1.67	2.98	2.40
		E	1.65	2.79	2.18	4.51	5.57	4.97	1.82	3.01	2.51
		S	1.62	3.03	2.36	4.61	5.64	5.19	1.81	3.20	2.51
					**2.23**			**5.09**			**2.47**
M. crocea	Ambohitantely	B	1.51	3.05	2.08	4.84	5.98	5.50	1.57	3.07	2.38
		E	1.85	3.21	2.43	5.04	6.09	5.60	1.85	3.14	2.47
		S	1.64	2.83	2.27	4.72	5.92	5.44	1.58	2.95	2.28
					**2.26**			**5.51**			**2.38**
	Ampangadimbolana	B	1.12	2.64	1.95	4.31	5.52	5.02	1.51	2.91	2.28
		E	1.53	2.81	2.13	4.20	5.42	4.83	1.78	3.01	2.32
		S	1.60	3.00	2.27	4.42	5.59	4.99	1.85	3.21	2.59
					**2.12**			**4.95**			**2.40**
M. milotympanum	Fierenana	B	1.44	2.89	2.18	4.44	5.55	4.96	1.51	2.81	2.17
		E	1.73	2.88	2.28	4.30	5.42	4.88	1.70	2.77	2.27
		S	1.57	2.87	2.21	4.41	5.56	5.01	1.50	2.77	2.05
					**2.22**			**4.95**			**2.16**
	Andriambe	B	1.36	2.74	2.06	4.26	5.45	4.90	1.56	2.91	2.17
		E	1.57	3.03	2.25	4.24	5.37	4.84	1.75	3.09	2.43
		S	1.40	2.96	2.11	4.39	5.55	4.91	1.69	3.03	2.47
					**2.14**			**4.88**			**2.36**
	Savakoanina	B	1.16	2.98	2.22	4.40	5.52	4.96	1.21	2.92	2.24

		E	1.75	3.20	2.53	4.23	5.33	4.74	1.67	2.96	2.32
		S	1.69	3.22	2.48	4.45	5.52	5.02	1.75	3.03	2.40
					**2.41**			**4.91**			**2.32**
